# The Nanopore-Tweezing-Based, Targeted Detection of Nucleobases on Short Functionalized Peptide Nucleic Acid Sequences

**DOI:** 10.3390/polym13081210

**Published:** 2021-04-09

**Authors:** Isabela S. Dragomir, Alina Asandei, Irina Schiopu, Ioana C. Bucataru, Loredana Mereuta, Tudor Luchian

**Affiliations:** 1 Sciences Department, Interdisciplinary Research Institute, Alexandru I. Cuza University, 700506 Iasi, Romania; isdragomir@yahoo.ro (I.S.D.); alina.asandei@uaic.ro (A.A.); iri.schiopu@gmail.com (I.S.); 2 Department of Physics, Alexandru I. Cuza University, 700506 Iasi, Romania; bucataru.cezara93@gmail.com (I.C.B.); loredana.mereuta@uaic.ro (L.M.)

**Keywords:** nanopore tweezer, peptide nucleic acid, sequencing, single-molecule recordings

## Abstract

The implication of nanopores as versatile components in dedicated biosensors, nanoreactors, or miniaturized sequencers has considerably advanced single-molecule investigative science in a wide range of disciplines, ranging from molecular medicine and nanoscale chemistry to biophysics and ecology. Here, we employed the nanopore tweezing technique to capture amino acid-functionalized peptide nucleic acids (PNAs) with α-hemolysin-based nanopores and correlated the ensuing stochastic fluctuations of the ionic current through the nanopore with the composition and order of bases in the PNAs primary structure. We demonstrated that while the system enables the detection of distinct bases on homopolymeric PNA or triplet bases on heteropolymeric strands, it also reveals rich insights into the conformational dynamics of the entrapped PNA within the nanopore, relevant for perfecting the recognition capability of single-molecule sequencing.

## 1. Introduction

Nucleic acid sequencing stands as the method of choice for revealing genetic variations at the molecular level, and it became undisputed in fundamental and clinical or forensic science, epidemiology, and biotechnology applications. The intrinsic limitations of approaches derived from or directly pertaining to the original Sanger sequencing method [1] include extensive and costly biochemical labeling, sample preparation, and difficulty to achieve long read lengths. To overcome this, single-molecule nanopore sequencing techniques that were “label-free” and relatively simple to operate and apply came to the rescue [2,3,4,5,6,7,8,9,10,11].

In the simplest embodiment, single-stranded DNA (ssDNA) is uni-directionally driven through an isolated nanopore, and it determines a characteristic ionic current blockade signature that can be used to infer the corresponding ssDNA sequence. In the late 1990s [12,13], a “proof-of-concept” demonstration of the approach was implemented with the α-hemolysin protein (α-HL) from *Staphylococcus aureus*, which formed large heptameric protein nanopores in lipid bilayers resembling a mushroom-like assembly with a central channel approximately 10 nm long and a diameter of 1.5 nm at the most constricted region [14].

Since then, massive creative efforts and the implication of other protein- or solid-state-based nanopores [7,9,15,16,17,18,19,20,21,22] have facilitated enormous leaps in the technique, bringing it closer to fulfilling the gold standard, which would enable a mammalian-sized genome to be sequenced for USD 1000 or less [23].

A pressing hindrance to achieving accurate nanopore sequencing is that ssDNA translocation is rapid, with measured rates of ~1 nt/μs at ∆V = 100 mV in the α-HL system [13,15], and this alone poses a serious challenge to the sensitive resolve of individual bases as they are driven through the nanopore. To time-extend the α-HL nanopore probing of individual single-stranded polynucleotide sequences, and to increase the signal-to-noise ratio of ionic current blockades occurring during translocation, various strategies were devised, including ssDNA ratcheting via the action of a DNA polymerase [24], a combination of exonuclease sequencing and an engineered α-HL pore equipped with a cyclodextrin molecular adapter [6,25], and immobilization of an ssDNA sequence within the nanopore via molecular “stoppers” [20,26,27,28,29,30].

In a previous related project from our lab, in order to enhance the time resolution of α-HL-based molecular detection and discrimination on polypeptides, we introduced a new method dubbed “the nanopore-tweezer approach”. In short, we used model polypeptides whose N- and C-termini were engineered to contain patches of glutamates and arginines, rendering them as macro-dipoles, and we demonstrated that an increase in the transmembrane potential (ΔV) led to an increase in both the polypeptide capture rate by the nanopore and the residence time inside the nanopore [31,32,33].

Since their discovery as structural DNA analogs containing an uncharged N-(2-aminoethyl) glycine-based pseudopeptide backbone and mimicking DNA by forming Watson–Crick complementary duplexes with normal DNA [34,35], charge-neutral peptide nucleic acids (PNAs) have demonstrated tremendous potential for antigene and antisense therapy, functional genomics, or as a probe, useful in the toolbox for diagnosis and detection [36,37,38,39,40,41,42,43,44,45], and constitute an excellent model substrate for devising innovative approaches directed at nucleic acids’ primary structure reading.

**Figure 1:**
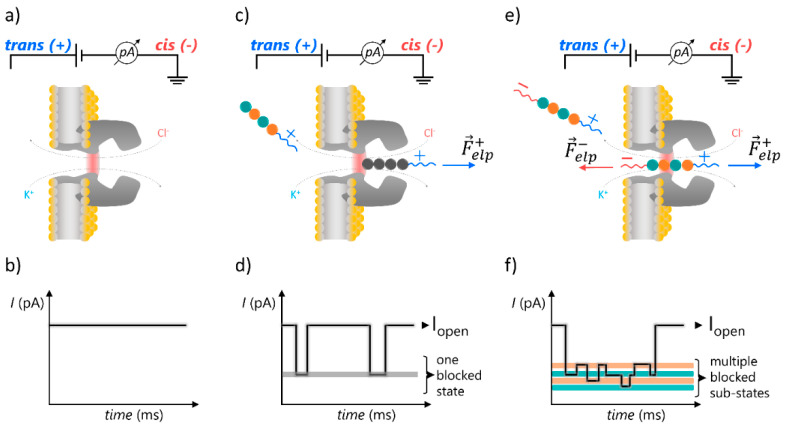
Simplified representation of the nanopore-tweezer technique, aimed at the primary structure characterization of individual PNAs. (**a**) In the absence of non-specific interactions, the ionic current through a single α-hemolysin protein (α-HL) nanopore isolated in a lipid membrane clamped at a constant potential difference (ΔV) remains constant (**b**). (**c**) Capture of an electrically charged analyte with the nanopore and its journey across the nanopore are seen as reversible changes of the ionic current through the nanopore between the open state (I_open_—free nanopore) and the blocked state (I_blocked_—nanopore transiently occupied by the analyte) (**d**). (**e**) If the PNA is decorated with oppositely charged segments at its ends, turning it into a macro-dipole during the capture events inside the voltage-biased nanopore, an electrostatic tug-of-war between opposite electric forces exerted at the sides of the analyte ensues, increasing the residence time of the analyte inside the nanopore. This allows for the visualization of characteristic ionic current fluctuations through the nanopore (**f**), whose features may correlate with the PNA’s primary structure.

Herein, we extended the α-HL nanopore-tweezer method and assessed the system’s ability to discriminate among distinct nucleobases on PNA sequences from ionic current fluctuations measured in a single PNA-α-HL blockade event. Our strategy was twofold: Firstly, we employed PNAs engineered with the lysine and glutamic acid segments at the N- and C-termini (Table 1). Their combined length was chosen to ensure that, while captured inside the α-HL nanopore in their unfolded form, such constructs fit inside the ~10-nm long α-HL pore, and the lysine and glutamic acids segments from the PNAs’ termini face the α-HL’s vestibule and α-barrel openings. In doing so, we sought to increase the construct’s mean residence time in the pore due to an electrostatic tug-of-war between the charges on opposite sides of the construct and the applied potential (Figure 1), as we demonstrated previously [31]. Secondly, all of the experiments were undertaken with the constructs added on the *trans* side of the membrane, in contact with the α-HL’s α-barrel, which was positively polarized with respect to the ground. Hence, we achieved an increased capture rate of the constructs at the α-barrel entry of the α-HL, as the net negative charges located at the nanopore entrance (~−7.3 |e^−^| at pH ~ 7.3) [46] decreased the free energy barrier for capture through attractive electrostatic interactions manifested between the PNAs—guided head-on towards the nanopore’s mouth with the lysine-containing terminus at positive ∆Vs—and the α-barrel [31,47].

## 2. Materials and Methods

### 2.1. Chemicals and Reagents

The polypeptide-functionalized PNAs used in this study (Table 1) were designed by us and synthesized and purified by Panagene Inc., Daejeon, Korea. The 1,2-diphytanoyl-sn-glycerophosphocholine lipid (DPhPC) was purchased from Avanti Polar Lipids, Alabaster, AL, USA, and the α-hemolysin monomeric protein (α-HL), potassium chloride (KCl), n-pentane, hexadecane, EDTA, and buffers (Tris(hydroxymethyl)aminomethane-Tris and 4-(2-Hydroxyethyl)piperazine-1-ethanesulfonic acid-HEPES) were procured from Sigma-Aldrich, Darmstadt, Germany.

### 2.2. Buffer Solutions and Sample Preparation

The 3-M KCl electrolyte solution used in the electrophysiology experiments was prepared in ultra-pure water and buffered with 10 mM HEPES at pH = 7.4. Stock solutions of 200 µM from the polypeptide-functionalized PNAs were made in 1 M NaCl, dissolved in ultra-pure water, buffered with TE (1 mM EDTA, 10 mM Tris) at pH = 8.25, and were kept at −20 °C before use. Preceding each experiment, the polypeptide-functionalized PNA solutions were heated to 95 °C using an IKA Digital Block Heater (Cole-Parmer, Vernon Hills, IL, USA) and slowly cooled down to ~23 °C. All experiments were performed at a room temperature of ~23 °C.

### 2.3. Electrophysiology Experiments

The lipid membranes for the electrophysiology experiments with nanopores were formed as described previously [48,49]. Insertion of a single α-HL protein nanopore in the bilayer membrane was achieved by adding small volumes of the protein solution in the grounded *cis* compartment of the bilayer chamber, followed by gentle stirring. The polypeptide-functionalized PNAs were added to the *trans* compartment from the stock solutions to achieve a final bath concentration of 9 µM. The PNA-induced fluctuations in the ionic current through the nanopore were recorded using two Ag/AgCl electrodes connected to an Axopatch 200B amplifier (Molecular Devices, CA, USA) set to voltage-clamp mode, at various holding voltages. Data acquisition was undertaken using an NI PCI 6221, 16-bit card (National Instruments, Austin, TX, USA) at a sampling frequency of 50 kHz and a low-pass filter at 10 kHz within the graphical programming environment LabVIEW 8.20 (National Instruments, Austin, TX, USA). The experimental set-up was shielded from environmental, electrical, and mechanical noise with a Faraday cage (Warner Instruments, Hamden, CT, USA) and mechanically isolated with a vibration-free platform (BenchMate 2210, Warner Instruments, Hamden, CT, USA). The all-amplitudes analysis of the ionic current fluctuations associated with the reversible α-HL-PNA interactions and Gaussian fitting of the resulting amplitude histograms were performed using Origin 6 (OriginLab, Northampton, MA, USA).

## 3. Results and Discussion

Encouraged by the successful application of the nanopore-tweezer technique for single-molecule interrogation of the primary structure on model polypeptides [50,51], we embarked, herein, on a “proof-of-concept” attempt to demonstrate bases’ recognition and discrimination on engineered PNAs with a similar approach (Figure 1).

By virtue of the previous geometrical considerations made within the frame of similar paradigms, whereby asymmetrically charge-tagged polypeptides were investigated with the α-HL nanopore [48,49], we posit that while captured inside the nanopore, the PNA’s middle domain bases most likely visit the nanopore’s constriction region (see schematics in Figure 1e). Having taken into account the constriction region dimensions (~0.6 nm in length, 1.4 nm in diameter, and an estimated volume of ~924 Å^3^) [14], and assuming that the current amplitude fluctuations associated with the presence of a PNA fragment inside the nanopore (Figure 1f) are chiefly correlated with the reversible blockade events occurring while the PNA slides back and forth along the sterically most sensitive region (i.e., the α-HL’s constriction region), a theoretical readout spatial resolution of ~1.6 bases on the PNA primary structure was proposed. Thus, central to the objective of reading the PNA sequence through such current recordings is the expectation that distinct blockade levels corresponding to specific bases presented in the nanopore’s constriction domain would permit their identification on a PNA sequence. It should be noted that such an approach has been previously validated by experimental results obtained with distinct protein nanopores [52,53].

## 4. Use of Homopolymeric PNAs to Investigate Sequence Recognition with the Nanopore

To examine the possibility of individual bases’ detection within a PNA chain, we designed distinct sequences comprising homopolymeric guanine, cytosine, adenine, and thymine (Table 1). As longer polyG strands cannot be readily synthesized due to the formation of secondary structures [54], the number of guanine bases was restricted to six.

**Figure 2:**
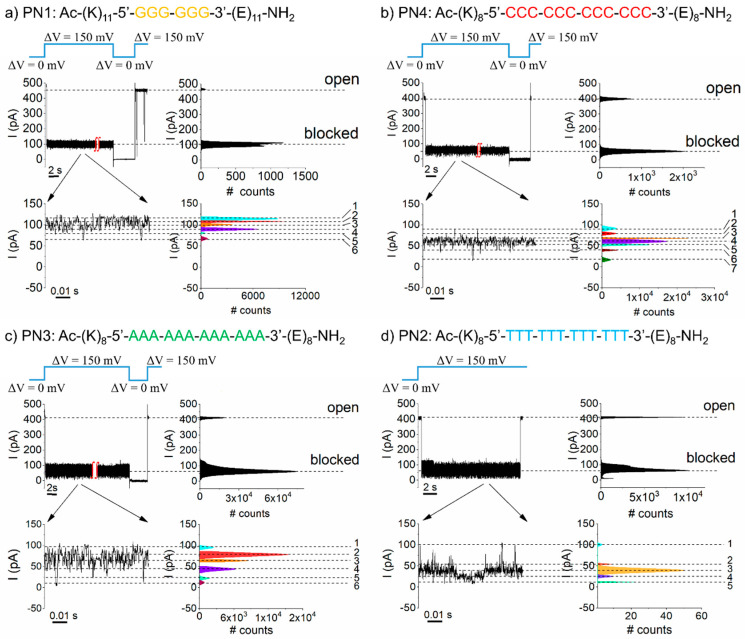
Discrimination of individual bases on homopolymeric PNAs. Selected traces illustrating the reversible blockades of the ion current through an open α-HL pore due to interactions with the (PN1) G_6_ (**a**), (PN4) C_12_ (**b**), (PN3) A_12_ (**c**), and (PN2) (T_12_) (**d**) PNAs. The all-points histogram on the right-hand side of each original trace illustrates the values of the average blockade level needed to calculate the total relative blockade of the nanopore (see, also, Table 2). All such experiments were carried out with PNAs added to the *trans* side of the membrane [9 μM], in an electrolyte containing 3 M potassium chloride (KCl) and 10 mM HEPES, pH = 7.4, and at a transmembrane potential of ∆V = +150 mV. In certain instances, the nanopore remained stuck in the “blocked” state upon capturing the PNA constructs; thus, a flip of the ∆V to 0 mV was required to dislodge the fragments from the nanopore. Close inspection of the residual currents through the PNA-blocked nanopores revealed supplementary blockade substates (zoomed-in excerpts on each panel), also quantified from the corresponding all-points histograms (see, also, Table 2).

In Figure 2, we represent selected traces demonstrating the reversible changes in the open-pore currents carried by the nanopore following interactions with distinct PNAs. In the simplest scenario, an entrapped macro-dipole-like PNA would position itself symmetrically around the constriction region of the nanopore. Based on the symmetry considerations, we posit that of the total of 6 (PN1 construct) to 12 bases (PN2, PN3, and PN4 constructs) present in the middle segment of constructs, nearly half of them (three bases—PN1 case—or six bases—PN2, PN3, and PN4 cases) most likely probed the constriction region and, partly, the adjacent half of the nanopore corresponding to the α-barrel, assigned to the first recognition site in the α-HL nanopore [55].

We sought base discrimination in terms of the differences in relative changes of the open nanopore current following fragments’ capture to the average “blocked” substate (denoted by total relative blockade—see Table 2). As a first finding, we noted that the order of total relative blockades $\left|\frac{I_{blocked}-I_{open}}{I_{open}}\right|$ corresponding to the average “blocked” substate, as shown in Figure 2, was C_12_ PNA ≈ T_12_ PNA > A_12_ PNA > G_6_ PNA. These results are in line with previous data demonstrating that poly(dA)60 oligonucleotides blocked the α-HL nanopore to a lesser extent than poly(dC)60, and at the proposed recognition site inside the nanopore closest to the constriction region (R1), also considered implicated herein, single thymine gave a larger block compared to adenine [55].

It should be reminded that, herein, unlike in previous related work, electrically neutral N-(2-aminoethyl) glycine repeating units in PNAs replaced the net negative sugar-phosphate backbone found in DNA so that the residual ionic current measured across the α-HL-PNA system was carried out by both anions and cations. This is relevant, as it has been proven that while captured inside the α-HL, charged analytes (dendrimers or ssDNAs) alter the ion selectivity the α-HL nanopore [29,56]. The molecular mechanism through which bases’ recognition by the α-HL is modulated by the PNA/ssDNA backbone charge and steric differences remains yet to be clarified.

In line with previous results from our laboratory, we noted the presence of additional PNA-induced conductance fluctuations of the α-HL, as the residual current measured across the α-HL-PNA system visited multiple substates (Figure 2a–d, zoomed-in traces in insets, and Table 1).

Remarkably, certain puzzling particularities still linger in the present work. While probing homopolymeric peptides with a similar system [50,51], we observed that the residual ionic current flipped randomly between only two distinct blockade substates, indicative of a simple model in which the deeper blockade corresponds to a group of three amino acids centered on the constriction region of the nanopore, while the shallower one was assigned to the same group of residues shifting out of the constriction region during the peptide passage across the nanopore.

Herein, such a relatively unambiguous interpretation is lacking, since, depending on the PNA studied, as many as 5 to 7 blockade substates were seen in the recorded trace (Figure 2). Although it is in stark contrast to our expectations (i.e., we predicted a similar blockade pattern of current fluctuations for the studied homopolymeric PNAs), one possible explanation for our results may lie in the stochastic nature of the disruptions in the conformational substates and the structure of the PNA within the nanopore [57,58], as it experiences fluctuating electric forces exerted at its oppositely charged moieties. This, in turn, would cause sterically related changes in the residual ionic current through the nanopore, seen as reversible fluctuations reported herein.

## 5. Triplet Base Recognition in a Heteropolymeric PNA Background

To further probe the PNA recognition by the α-HL nanopore, constructs presenting alternated triplet bases in the middle domains were proposed. Based on their individual volumes (V_C_ = 115 Å^3^, V_T_ = 138 Å^3^, V_A_ = 139.2 Å^3^, and V_G_ = 145.9 Å^3^) [59], and to generate heteropolymers able to affect, with the greatest propensity, the ionic current across the nanopore as a result of bases’ substitutions, we designed sequences containing two consecutive alternating groups of guanine and thymine in the middle section, namely PN5 (K_8_–G_3_–T_3_–G_3_–T_3_–E_8_) and PN6 (K_8_–T_3_–G_3_–T_3_–G_3_–E_8_), respectively (Table 2).

**Figure 3:**
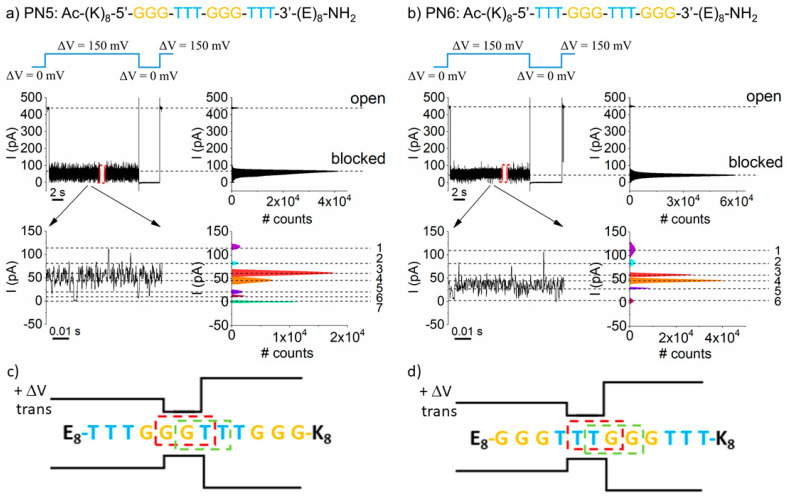
The influence of the PNAs’ primary structure on the ionic current fluctuations. Representative current recordings through a single α-HL nanopore clamped at ∆V = + 150 mV displaying the transient pore blockades by (**a**) PN5 (K_8_–G_3_–T_3_–G_3_–T_3_–E_8_) and (**b**) PN6 (K_8_–T_3_–G_3_–T_3_–G_3_–E_8_) PNAs added to the *trans* side of the lipid membrane [9 μM], in an electrolyte containing 3 M KCl and 10 mM HEPES, pH = 7.4. The additional fluctuations of the residual ionic current are presented in the zoomed-in excerpts, together with the corresponding all-points histograms showing the amplitude distribution of the blockade substates. The sketches in (**c**) and (**d**) help to illustrate the orientation of the distinct triplet bases from the PN5 and PN6 constructs most likely occluding the α-HL’s constriction region during the meta-stable capture events (see text). The drawn α-HL displaying the α-barrel (*trans* side), constriction region, and vestibule opening is not shown to scale.

The representative data shown in Figure 3 indicate that the order of bases in the PNAs’ primary sequence influenced the total relative blockade describing the average “blocked” substate (Table 2), as well as the residual current fluctuations seen within the "blocked” substate, in terms of substate number, amplitude, and relative occupancies, as judged qualitatively from the distribution of Gaussian peaks in the all-points histograms (see, also, Table 2). This, in turn, was unexpected, as in either case (i.e., the nanopore transiently blocked by PN5 (K_8_–G_3_–T_3_–G_3_–T_3_–E_8_) or PN6 (K_8_–T_3_–G_3_–T_3_–G_3_–E_8_) PNA), a similar heterogeneous frame of three bases out of the overall available pool—namely, either GGT, GTT, TTG, or TGG—presented itself and was “read” at the α-HL’s constriction region at a given time. In other words, regardless of the PNA type (either PN5 or PN6), a similar number of blockades were predicted to ensue during a single PN5 or PN6 capture.

To account for the heterogeneity of the blockade substate distributions recorded, one must recall that by virtue of the detection principle implicated herein (vide supra) and geometrical considerations [48], an entrapped PN5 or PN6 construct presents, with the largest likelihood, its middle section near the nanopore’s constriction region. Knowing that at + ∆Vs, both *trans*-added PN5 and PN6 constructs enter the nanopore’s α-barrel with the (K)_8_ residues head-on (Figure 3c,d), it follows that the distinct triplet bases “read” at the constriction region are GGT or GTT (PN5 construct, Figure 3c) and TTG or TGG (PN6 construct, Figure 3d), respectively. This suggests that the uneven distribution of blockade levels within the residual ionic current in Figure 3 are correlated with the distinct base triplets read by the nanopore in either case.

## 6. The PN6 (K_8_–T_3_–G_3_–T_3_–G_3_–E_8_) PNA-Induced Conductance Fluctuations in a Single α-HL Nanopore are Voltage-Dependent

**Figure 4:**
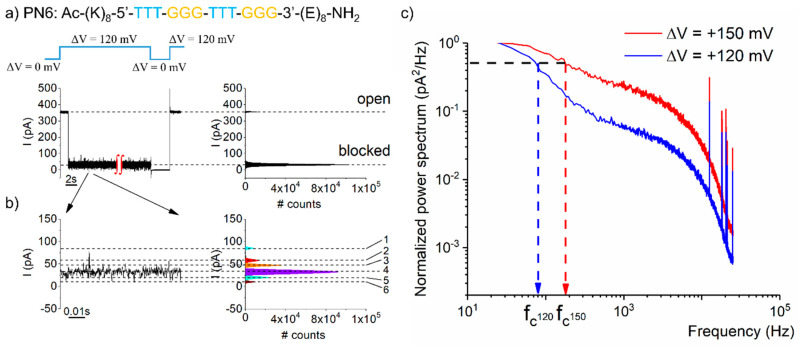
The fluctuation kinetics of the residual current through the α-HL-PNA system were voltage-dependent. (**a**) Excerpted segment from a recording made at ∆V = +120 mV, capturing a PN6 (K_8_–T_3_–G_3_–T_3_–G_3_–E_8_) PNA-induced blockade event on the ionic current mediated by the α-HL. The expanded trace in (**b**) displays the fluctuations making up the residual current through the α-HL while blocked by the PNA. (**c**) Representative normalized power spectra of residual current fluctuations entailed by a PN6 PNA lodged inside the α-HL, recorded at two distinct potentials.

In the previous chapter, “Use of homopolymeric PNAs, to investigate sequence recognition with the nanopore”, we postulated that the current fluctuations seen while a PNA fragment is lodged within a nanopore may reflect the dynamic unfolding of distinct conformational substates of the PNA within the nanopore. To verify this assertion, we recorded and analyzed the kinetics of such fluctuations seen with the PN6 (K_8_–T_3_–G_3_–T_3_–G_3_–E_8_) PNA heteropolymer entrapped inside the α-HL (Figure 3b and Figure 4) at two distinct ∆Vs.

While the amplitude distribution of the residual blockade ionic current recorded at ∆V = +120 mV suggested a similar number of six blockade substates (Figure 4b), as measured at +150 mV (Figure 3b), the kinetics of such fluctuations were faster in the latter case (Figure 4c). For brevity, we quantified the “corner frequency” (f_c_) of the power spectra generated at the two ∆Vs: f_c_(∆V = +120 mV) = 76.2 ± 11.7 Hz, and f_c_(∆V = +150 mV) = 132.2 ± 41.4 Hz.

In relation to the hypothesis made to explain the molecular mechanism underlying such fluctuations, we suggest that a larger electric force acting on the entrapped PNA, entails a pronounced disruption of stacking interactions [60], thus altering the kinetic behavior and folding conformations of the molecule within the nanopore. Alternatively, one could propose additional contributions stemming from the voltage-dependent movement fluctuations of the studied PNA fragments inside the nanopore. For our case, this seems counter-intuitive, as the tug-of-war between the forces acting at the ends of the oppositely charged PNA not only stabilizes the entrapped molecule, but elevated forces—manifested at larger ∆Vs—would deepen the central minimum in the free-energy profile of the entrapped PNA and further stabilize it [31,32]. An elevated level of understanding of these phenomena, which is extremely relevant for the task of polymers sequencing with nanopores, requires further experimental and theoretical refinement.

## 7. Conclusions

To further expand the paradigm of nanopores’ suitability for single-molecule sequencing applications, we employed, in the present work, the α-HL protein nanopore in conjunction with the nanopore tweezing technique and demonstrated its ability to provide a base-specific readout on model PNAs. It was shown that the nanopore system can recognize bases in homopolymeric PNA, and the single-molecule stretching experiments of PNA strands inside the nanopore revealed fluctuations of the residual current, which may reflect the fact that the studied PNAs adopt multiple conformations as they translocate through the nanopore, thus distinctly altering the nanopore conductance. Although qualitative in nature, with further experimentation strategies (e.g., nanopore mutagenesis, site-directed chemical modification and variable PNA composition, temperature, and salt concentrations), our findings may provide powerful diagnostics for the coupling of stacking interactions with the elastic properties of individual nucleic acid fragments, complementary to established protocols [61]. We also discovered that the order of T and G bases in the triplets probing the nanopore’s constriction region generated pronounced differences in the residual current fluctuations through the nanopore. Although the total blockade currents measured in α-HL arising from nucleotides were not uniquely attributable to an individual base in a specific position, our findings are consistent with recently published studies in which we demonstrated that α-HL sensitivity to molecular exclusion at the most constricted region provides the specificity needed to discriminate between distinct groups of amino acids [48,49].

## Figures and Tables

**Table 1:** The primary structure of the polypeptide-functionalized peptide nucleic acids (PNAs) and their corresponding generic names employed herein.

**PN1**	Ac-(K)_11_-5′-GGG-GGG-3′-(E)_11_-NH_2_
**PN2**	Ac-(K)_8_-5′-TTT-TTT-TTT-TTT-3′-(E)_8_-NH_2_
**PN3**	Ac-(K)_8_-5′-AAA-AAA-AAA-AAA-3′-(E)_8_-NH_2_
**PN4**	Ac-(K)_8_-5′-CCC-CCC-CCC-CCC-3′-(E)_8_-NH_2_
**PN5**	Ac-(K)_8_-5′-GGG-TTT-GGG-TTT-3′-(E)_8_-NH_2_
**PN6**	Ac-(K)_8_-5′-TTT-GGG-TTT-GGG-3′-(E)_8_-NH_2_

**Table 2:** Relative blockage values $\frac{I_{blocked}-I_{open}}{I_{open}}$ calculated for the average “blocked” substate (total relative blockade), as well as additional substate blockades seen in the zoomed-in traces in Figure 2 and Figure 3, denoted by corresponding numbers, at ∆V = + 150 mV.

	**PN1: K_11_ − G_6_ − E_11_**	**PN4: K_8_ − C_12_ − E_8_**
Total relative blockade	*−0.746 ± 0.007*	*−0.849 ± 0.012*
1	−0.735 ± 0.002	−0.779 ± 0.007
2	−0.756 ± 0.007	−0.813 ± 0.003
3	−0.779 ± 0.003	−0.839 ± 0.003
4	−0.800 ± 0.002	−0.857 ± 0.001
5	−0.817 ± 0.003	−0.873 ± 0.002
6	−0.862 ±0.011	−0.901 ± 0.005
7	-	−0.941 ± 0.005
	**PN3: K_8_ − A_12_ − E_8_**	**PN2: K_8_ − T_12_ − E_8_**
Total relative blockade	*−0.830 ± 0.023*	*−0.844 ± 0.034*
1	−0.736 ± 0.005	−0.701 ± 0.009
2	−0.791 ± 0.005	−0.760 ± 0.012
3	−0.82 ±0.002	−0.846 ± 0.003
4	−0.872 ± 0.004	−0.940 ± 0.006
5	−0.943 ± 0.002	−0.97 ± 0.014
6	−0.973 ± 0.001	-
	**PN5: K_8_ − (G_3_ − T_3_)_2_ − E_8_**	**PN6: K_8_ − (T_3_ − G_3_)_2_ − E_8_**
Total relative blockade	*−0.839 ± 0.021*	*−0.871 ± 0.007*
1	−0.742 ± 0.004	−0.748 ± 0.005
2	−0.809 ± 0.003	−0.822 ± 0.001
3	−0.858 ± 0.003	−0.875 ± 0.003
4	−0.898 ± 0.004	−0.901 ± 0.002
5	−0.943 ± 0.002	−0.95 ± 0.001
6	−0.978 ± 0.001	−0.988 ± 0.001
7	−0.996 ± 0.0005	-

## Data Availability

The data presented in this study are available on request from the corresponding author.
